# Factors influencing estimates of coordinate error for molecular replacement

**DOI:** 10.1107/S2059798319015730

**Published:** 2020-01-01

**Authors:** Kaushik S. Hatti, Airlie J. McCoy, Robert D. Oeffner, Massimo D. Sammito, Randy J. Read

**Affiliations:** a Department of Haematology, Cambridge Institute for Medical Research, University of Cambridge, Cambridge, England

**Keywords:** molecular replacement, coordinate error, root-mean-square deviation, r.m.s.d., NMR, log-likelihood gain, LLG

## Abstract

Good prior estimates of the effective root-mean-square deviation (r.m.s.d.) between the atomic coordinates of the model and the target optimize the signal in molecular replacement, thereby increasing the success rate in difficult cases. Previous studies using protein structures solved by X-ray crystallography as models showed that optimal error estimates (refined after structure solution) were correlated with the sequence identity between the model and target, and with the number of residues in the model. Here, this work has been extended to find additional correlations between parameters of the model and the target and hence improved prior estimates of the coordinate error. Using a graph database, a curated set of 6030 molecular-replacement calculations using models that had been solved by X-ray crystallography was analysed to consider about 120 model and target parameters. Improved estimates were achieved by replacing the sequence identity with the Gonnet score for sequence similarity, as well as by considering the resolution of the target structure and the *MolProbity* score of the model. This approach was extended by analysing 12 610 additional molecular-replacement calculations where the model was determined by NMR. The median r.m.s.d. between pairs of models in an ensemble was found to be correlated with the estimated r.m.s.d. to the target. For models solved by NMR, the overall coordinate error estimates were larger than for structures determined by X-ray crystallography, and were more highly correlated with the number of residues.

## Abstract 2

Improved coordinate error estimates are proposed for the X-ray and NMR models used for maximum-likelihood-based molecular-replacement phasing.

## 1. Introduction   

Likelihood-based molecular replacement (MR) uses estimates of the errors in the model and the data to improve the signal to noise in the search. In *Phaser* (McCoy *et al.*, 2007 ▸), the log-likelihood gain on intensities (LLGI; Read & McCoy, 2016 ▸) accounts for the effect of intensity measurement errors when scoring MR searches. The LLGI discriminates correct from incorrect solutions and is used to rank solutions across complex search strategies (Oeffner *et al.*, 2018 ▸), such as those implemented in the *ARCIMBOLDO* suite of programs (Millán *et al.*, 2015 ▸), *AMPLE* (Rigden *et al.*, 2008 ▸; Bibby *et al.*, 2013 ▸) and *MrBUMP* (Keegan & Winn, 2008 ▸).

The LLGI (for acentric reflections) is defined as 
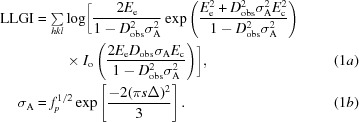



In this equation, the parameters *E*_e_ (effective *E*) and *D*_obs_ (Luzzati-style *D* factor) are derived from the measured intensity and its estimated standard deviation (Read & McCoy, 2016 ▸), resulting in any reflections with large experimental errors being downweighted. This gives an excellent approximation to an intensity-based likelihood target that would require expensive numerical integration. The σ_A_ term accounts for the effect of predicted errors in the model. LLGI calculations will be optimal when the initial estimates of σ_A_ are accurate. Underestimation of σ_A_ will lead to underweighting of the high-resolution reflections in the LLGI calculations, whereas overestimation of σ_A_ will lead to overweighting of these reflections. Both problems will lead to the suboptimal usage of data and can influence success in a borderline case.

Ignoring an optional bulk-solvent term for simplicity, σ_A_ can be expressed as a function of resolution (*s* = 1/*d*), model completeness (*f_p_*, the fraction of total scattering accounted for by the model) and the effective r.m.s. coordinate error of the model (Δ) as given in (1*b*). Once the model has been placed in the MR calculation, the value of Δ can be refined during a rigid-body refinement. This term Δ is different from the r.m.s.d. that can be calculated between equivalent atomic positions by superposing two structures, because it is an effective r.m.s.d. that optimizes the variance term in the LLGI target. For this reason, we refer to it as variance-r.m.s.d. or, for short, VRMS.

The VRMS can only be refined once a model has been placed and its value is only relevant if the model is placed correctly, so it is necessary to provide a prior estimate of the VRMS before carrying out the search. Prior to *Phaser* v.2.5.4, *Phaser* used the Chothia and Lesk curve (which relates the sequence identity to the r.m.s.d. between main-chain atoms; Chothia & Lesk, 1986 ▸) as a first-order approximation. Although these values worked reasonably well, it became clear that estimates tailored to the MR problem were needed. We developed an improved functional form to estimate VRMS (2) as a function of the size of the model (*N*_res_) and the sequence identity (*H*, the fraction of mutated residues) between the model and the target (Oeffner *et al.*, 2013 ▸):
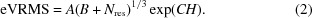



However, experience using a wide variety of MR models has shown that sequence identity is a poor measure to assess the sequence similarity of very distant homologues. We considered a number of alternative sequence-similarity measures that have been developed over the past few decades and that are summarized very well by Vogt *et al.* (1995 ▸).

To assess which property might improve predictive power, we also investigated the correlations of a variety of properties of the model and the target with the refined VRMS term. Because work up to this point had concentrated on models derived by X-ray crystallography, we also developed a new functional form to estimate VRMS specifically for members of NMR ensembles used as phasing models.

## 2. Methodology   

The study follows the methods described by Oeffner *et al.* (2013 ▸). Here, we summarize the steps from Oeffner and coworkers that were used to carry out large-scale molecular-replacement trials for X-ray models. The extension of the earlier work to include NMR models is elaborated below.

### 2.1. Generation of molecular-replacement data using X-ray models   

In the earlier study, a total of 2862 structures (and the associated diffraction data) with a single chain in the asymmetric unit, across a range of SCOP classes (Murzin *et al.*, 1995 ▸) and with a size varying between 50 and 1500 residues, were selected as targets from the wwPDB (Berman *et al.*, 2000 ▸). Care was taken not to include targets that were known to be twinned or for which the published *R* factors could not be reproduced by the Uppsala Electron Density server (Kleywegt *et al.*, 2004 ▸). Only one example was kept for each unique sequence, except that all entries for proteins with more than 600 residues were retained to improve the sampling of large targets. For each target, homologous structures were identified by performing a *BLAST* search (Altschul, 1991 ▸) against the wwPDB with the *BlastP* tool. *ClustalW* (Thompson *et al.*, 1994 ▸) was used to perform pairwise alignments of the homologue and target sequences; unlike *BLAST*, which finds local sub­sequence alignments, *ClustalW* maximizes the global sequence alignment. The models were pruned and edited with *Sculptor* (Bunkóczi & Read, 2011*a* ▸). A total of 21 822 molecular-replacement calculations were performed and used for analysis in the earlier study.

For this study, we curated the database from the earlier study to remove redundant targets (inadvertently included more than once) and models that failed to lead to successful molecular-replacement solutions. To measure the reliability of the molecular-replacement solution, we calculated model-to-map correlations (globalCC) using *phenix.get_cc_mtz_pdb* to assess the agreement between 2*mF*_o_ − *DF*_c_ maps (Read, 1986 ▸) computed from the molecular-replacement solution and the deposited model. A subset of 6030 molecular-replacement trials with globalCC > 0.2 was chosen, in the end, from the curated database. These trials arise from a combination of 1307 distinct targets (which include 119 targets with deposited intensity data) and 3420 distinct models. The database was extended to include a variety of parameters associated with target, model and sequence-similarity measures.

#### 2.1.1. Target properties   

Several measures to assess crystal parameters, data parameters and protein parameters were downloaded from the wwPDB. See Table 1 ▸ for a complete list of target properties considered in the study.

#### 2.1.2. Model properties   

Parameters such as the number of residues, date of deposition, resolution, r.m.s. deviations of bond lengths and angles from ideal values and *R* factors were downloaded from the wwPDB. Validation parameters such as Ramachandran properties, clashscore, rotamer outliers, *MolProbity* score (Chen *et al.*, 2010 ▸) and C^β^ deviations were recalculated for the processed models using *Phenix* (Liebschner *et al.*, 2019 ▸) command-line tools. Nonsphericity of the model was estimated by calculating principal axes using *Gromacs* (Abraham *et al.*, 2015 ▸) command-line tools.

When available, SCOP definitions were downloaded from the SCOPe database (Fox *et al.*, 2014 ▸) and assigned to both target and model entries (Table 1 ▸).

#### 2.1.3. Sequence-similarity properties   

Several amino-acid substitution matrices were used to assess the sequence similarity of a target–model pair. In this study, we considered matrices that were judged to assess sequence similarity accurately for pairwise sequence identities below 50% (Vogt *et al.*, 1995 ▸; Table 1 ▸). The matrices were used from within *Biopython* (v.1.72) to score every target–model pairwise sequence alignment. The scores were normalized for the length of aligned residues.

### 2.2. Generation of molecular-replacement data using NMR models   

A protocol similar to that used to generate molecular-replacement data with X-ray models was used for the NMR models. The targets identified above were retained and no new targets were considered for this study.

#### 2.2.1. Selection of NMR models   

The sequence-profile database constructed using entries from the PDB at 70% sequence nonredundancy, PDB_mmCIF70, was downloaded from the *HHpred* (Zimmermann *et al.*, 2018 ▸) website. For a given target (as selected previously in Section 2.1) *HMMER* (Finn *et al.*, 2011 ▸) was used to identify homologous structures from PDB_mmCIF70. 1364 homologous structures which were determined by NMR alone were retained. Properties specific to NMR models such as the number of models deposited in an ensemble and chemical shift data validation were downloaded from the wwPDB (if reported).

#### 2.2.2. Processing of NMR models   

*Clustal Omega* (Sievers *et al.*, 2011 ▸), an improved implementation of the *Clustal* algorithm, was used to perform pairwise alignment of target and NMR model sequences. The scores discussed for X-ray models were also used to evaluate sequence similarity for NMR models. Models were pruned and edited with *Sculptor* (Bunkóczi & Read, 2011*a* ▸). Other studies have shown that using NMR models for MR phasing is a challenge and have suggested trimming protocols to improve success in molecular-replacement phasing (Chen *et al.*, 2000 ▸; Mao *et al.*, 2011 ▸). Accordingly, ensembles were generated with *Ensembler* (Bunkóczi & Read, 2011*b* ▸), selecting the default option to trim residues deviating by more than 3 Å. *Gesamt* (Krissinel, 2012 ▸) was used to perform a pairwise combination superposition of all versus all trimmed models in an NMR ensemble. A median r.m.s.d. between equivalent C^α^ positions was calculated for each trimmed ensemble to assess the conformational differences among the models. See Table 1 ▸ for the list of NMR-specific metrics considered in this study.

#### 2.2.3. Molecular-replacement rigid-body refinement   

NMR models with over 50% sequence coverage were superposed onto the target using *Gesamt*. A total of 20 973 molecular-replacement rigid-body refinements was performed using the MR_RNP mode of *Phaser* (McCoy *et al.*, 2007 ▸) using each model from the trimmed NMR ensemble independently. In practice, it is best to use NMR models as ensembles, but success in statistical weighting of the ensembles depends on having the best estimate of the effective error of each individual member of the ensemble (Read, 2001 ▸).

### 2.3. Generation of graph database   

**Figure 1:**
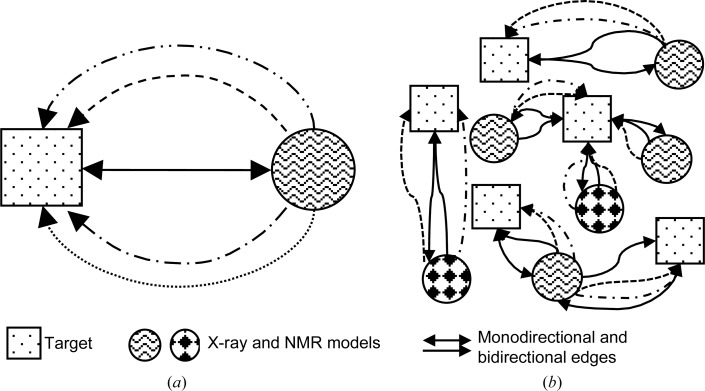
Schematic representation of the graph database. Targets and models are represented as square and circular nodes, while an edge connecting two nodes represents a relationship between a target and a model node. (*a*) Two types of edge can connect a target–model pair. (i) A unidirectional edge defines a single instance of a molecular-replacement trial in which a model was used to determine the target structure. The four different unidirectional edges represent four different trials of molecular replacement, for instance using data to different resolution limits. (ii) A bidirectional edge defines properties associated with sequence-similarity measures. More than one unidirectional edge exists between a target–model pair if more than one molecular-replacement trial was carried out. (*b*) presents an overview of a small graph database to show interconnections between the nodes. A single PDB entry could be used to determine two different targets; in which case the properties associated with processing the model, such as the *MolProbity* score of the processed model, are stored as part of the edge property. There are also examples where a single target could be determined using multiple independent models.

For a given pair of target and model, there were about 120 properties to be evaluated. To address this large-scale comparison, we built an in-house database representing the data as a graph, using the open-source graph database platform *Neo*4*j* (v.3.4.0; https://neo4j.com). The target and model were defined as nodes and an edge connecting the two defined a relationship (Fig. 1 ▸ *a*). All of the properties associated with a target or a model were associated with their respective nodes. Properties such as sequence-similarity scores and the results of molecular-replacement calculations were associated with the edge connecting the two nodes. In this way, a complex graph network was generated, which included all of the data defining the targets, models (both X-ray and NMR) and the relationships between them (Fig. 1 ▸ *b*). An intermediary layer of nodes (not shown in Fig. 1 ▸ for the sake of clarity) was used to represent model number in the case of NMR ensembles. Cypher, a declarative graph-querying language, was used to query the data.

All statistical analysis was performed within the *R* statistical programming environment (*R* v.3.5.0; R Core Team, 2018 ▸). Nonlinear least-squares fitting was performed using the *nls* package (Baty *et al.*, 2015 ▸) starting with the most highly correlated parameter and subsequently adding more parameters until a low residual correlation with unused parameters was obtained. Figures were generated using the *ggplot*2 package (Wickham, 2016 ▸). Both the *nls* and *ggplot*2 packages are available within *R*.

### 2.4. Derivation of equations to predict the refined VRMS   

In fitting the two data sets, the data were examined to determine which properties were most highly correlated with the refined VRMS. In general, one property was included at a time. Different functional forms were tested for equations adding that property when fitting to the data, and the functional form that minimized the deviation between the refined and estimated VRMS was chosen. To choose the next property to include in the fit to the data, residual correlations (correlation to the normalized difference between the refined and estimated VRMS) were computed. The process was terminated when adding a new property had little effect on the quality of the fit.

## 3. Results   

### 3.1. Improved estimates for X-ray models   

The Gonnet matrix score (Gonnet *et al.*, 1992 ▸) has the highest correlation to the refined VRMS term (Table 2 ▸) among all of the metrics used to estimate sequence similarity, so this was chosen to play the role taken by sequence identity in equation (2) from Oeffner *et al.* (2013 ▸). Among the properties of the model, the size of the model has the highest correlation to VRMS, followed by the *MolProbity* score. As judged by the residual correlation (also shown in Table 2 ▸), the *MolProbity* score was the most significant model feature that had not been considered in the work by Oeffner *et al.* (2013 ▸). Although we had only expected properties involving the model to play a significant role, we found target resolution to also correlate with VRMS, with a higher correlation than the *MolProbity* score (Table 2 ▸). Further molecular-replacement calculations were performed to ascertain that the correlation is not an artefact of the resolution of the data used during the VRMS refinement. Molecular-replacement calculations were repeated as a function of the target resolution by truncating the data to lower resolution limits (2.2, 2.7, 3.0, 3.5, 4, 6 and 7 Å), only to find that the correlation between VRMS and the original resolution of the target persisted.

Different functional forms for a nonlinear least-squares fit to the data from the 6030 molecular-replacement trials in the curated database were tested in preliminary work, including sums and products involving different properties and different choices of exponent for terms related to particular properties. The best results were obtained using equations expressing the total variance as a sum of independent variance terms.

**Figure 2:**
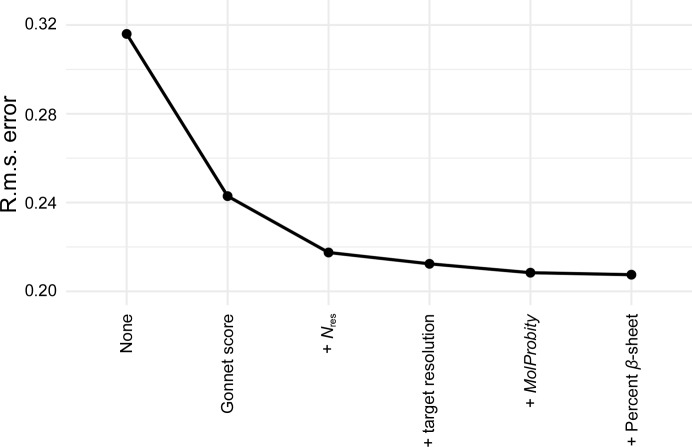
R.m.s. error in estimated VRMS as new properties are added to the prediction. Before any properties had been included (‘None’), the r.m.s. error was the r.m.s. deviation of the refined VRMS values from their mean for all calculations.

Fig. 2 ▸ shows the effect of including successive variance terms. Diminishing returns were achieved as new properties with lower explanatory power were added. After the *MolProbity* score had been included, the most significant remaining property was the percentage of β-sheet in the model, with a residual correlation of −0.13. However, including this property in the nonlinear fit had very little effect on the quality of fit, so it was not included in the final equation (3). Note that much of the correlation with α-helix content had apparently been accounted for by this point by correlations with other properties.
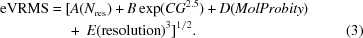



**Figure 3:**
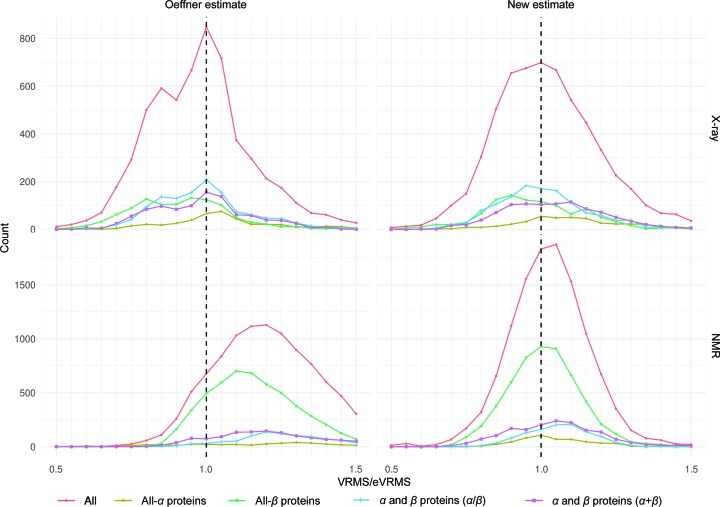
Frequency distribution of refined over estimated VRMS ratios from the curated data set as a function of SCOP class. A red line represents all cases. An ideal distribution should be Gaussian, with the lowest possible variance, and centred on 1 (represented by a black dashed line). X-ray case: the Oeffner estimate has a shoulder, which is not present in the new X-ray estimate. NMR case: the distribution for the Oeffner estimate based on X-ray data is shifted to the right, indicating that errors are systematically underestimated when applied to models derived by NMR. The new estimate based on NMR data has a symmetrical distribution centred around 1.

The nonlinear least-squares fit of (3) yielded the coefficients *A* = 0.001455, *B* = 1.710, *C* = −0.2444, *D* = 0.1040, *E* = 0.01586. Residual correlations computed using the new expression for eVRMS show that this functional form accounts for most of the initial systematic variation in the data (Table 2 ▸). In addition, a frequency distribution computed from the ratios of estimated and refined VRMS values became more symmetrical and unimodal than using the previous Oeffner coordinate error estimate (Fig. 3 ▸).

Fig. 3 ▸ also shows that the VRMS distributions are slightly different for different SCOP fold classes, with errors being slightly underestimated on average for all-α proteins and slightly overestimated for all-β proteins. However, in keeping with the very minor effect on the fit of including percentage β-sheet content, the differences in the distributions for fold classes are small compared with the width of the overall distribution.

### 3.2. Estimates for NMR models   

Previously published work (Chen *et al.*, 2000 ▸) and anecdotal evidence suggested that models obtained using NMR data generally work more poorly in MR than models obtained using X-ray data. In addition, we anticipated that a different functional form might be needed to predict model quality. For instance, considering that NMR structures are defined primarily by short-range distance data, one might expect an increased dependence of coordinate error on model size. In addition, NMR structures are usually reported as an ensemble of alternative models (typically 20) that all have a comparable fit to the data, and one might expect the deviation among these models to provide an indication of model precision, if not accuracy. Indeed, the analysis of correlations revealed that for NMR models there was a stronger correlation between refined VRMS and model size than for X-ray data, and there was a significant correlation with the deviation among the models in the ensemble (Table 3 ▸).

We wanted to check whether the estimates for NMR models could be improved by including criteria recommended by the NMR validation task force (Montelione *et al.*, 2013 ▸). For example, completeness refers to the percentage of chemical shifts that have been assigned. Surprisingly, no correlation was found between this completeness measure and VRMS. Other measures were reported only for a fraction of the NMR models included in this study and hence could not be studied further. It may be worth revisiting this analysis when larger numbers of NMR structures report these validation metrics.

A new functional form, given in (4), was defined, again estimating the overall variance as a sum of independent variance contributions and testing different exponents for the underlying variables. The quality of fit was only weakly affected by the exponent for the *N*_res_ term, probably because the range of model sizes is limited for NMR models. Unexpectedly, an exponent of 1/3 was slightly better than the exponent of 1 found for the X-ray fit; even though VRMS is more sensitive to model size for NMR compared with X-ray models, this sensitivity comes from the multiplicative factor *A* rather than the exponent.
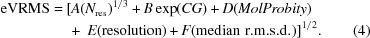



The six parameters in this equation were fitted using a subset of 12 610 molecular-replacement cases (with globalCC > 0.2) where NMR structures were used as models, limiting the data to structures that were between 30 and 300 residues in length. The *MolProbity* score for (4) corresponds to the individual *MolProbity* score of each model in a given NMR ensemble. The median r.m.s.d. is the median of the r.m.s.d.s of all pairwise superpositions of members of a given NMR ensemble. The nonlinear least-squares fit yielded the coefficients *A* = 0.4240, *B* = −1.259, *C* = 0.07804, *D* = 0.1442, *E* = 0.2364, *F* = 0.4130. All residual correlations were close to zero, giving a substantial improvement over the Oeffner estimates derived from X-ray models (Table 3 ▸).

### 3.3. The importance of accurate VRMS estimates   

It is important to start the calculations with accurate estimates of VRMS to achieve the highest initial LLGI scores, because the absolute value of the LLGI score is highly correlated to the signal to noise achieved in the search (McCoy *et al.*, 2017 ▸). To evaluate this, we calculated the LLGI in rigid-body refinements starting with the correctly placed model but without refining the VRMS parameter. The same set of cases used for curve fitting of both X-ray and NMR models were considered in this study. The calculations using both X-ray-derived and NMR-derived models were performed with both the Oeffner and the new estimates of VRMS. For NMR models, only the first member of the NMR ensemble was considered in these calculations.

**Figure 4:**
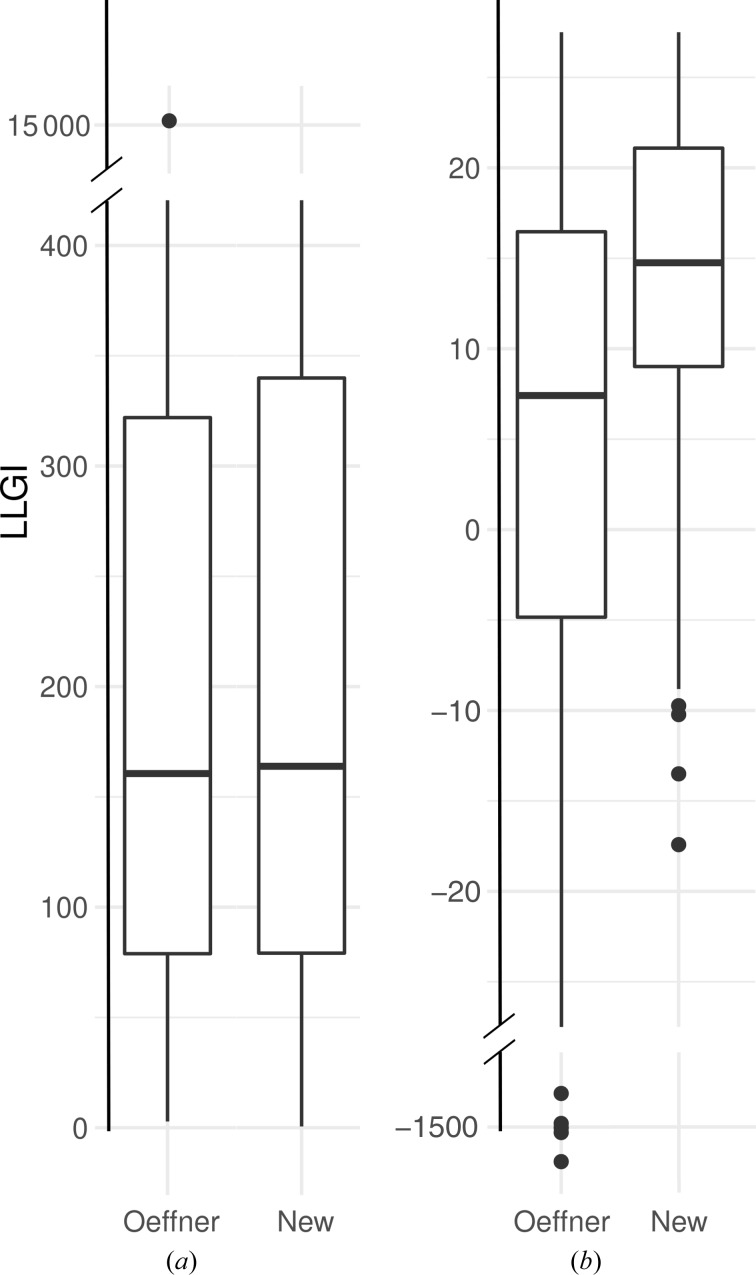
Calculation of LLGI starting with the Oeffner and new estimates of VRMS performed without VRMS refinement. (*a*) Values for X-ray models. (*b*) Values for NMR models. A limited range of LLGI values (along with the most extreme outliers) is displayed for the sake of clarity.

An incremental improvement was observed in the case of the X-ray models. The LLGI calculated with the new VRMS estimates (median LLGI = 163.9) was slightly better than that calculated with Oeffner estimates (median LLGI = 160.1) (Fig. 4 ▸). However, a larger improvement was observed in the case of the NMR models, where the median LLGI was 7.4 for calculations using the Oeffner estimates based on X-ray models and 14.7 using the new values derived for NMR models. The distribution of LLGI values for the NMR models has also become much narrower using the new VRMS estimates (Fig. 4 ▸). Note that few NMR models in our tests yield an LLGI score of 60 or more, which would normally indicate a correct solution, but the new LLGI values have been brought into a range that should help to enrich a pool of potential solutions with correct solutions (McCoy *et al.*, 2017 ▸). It should be noted that the calculations reported here used individual NMR models in order to calibrate the VRMS estimates, but in a real molecular-replacement search one would use the whole ensembles, which would improve the results.

### 3.4. Comparative analysis of X-ray and NMR models   

**Figure 5:**
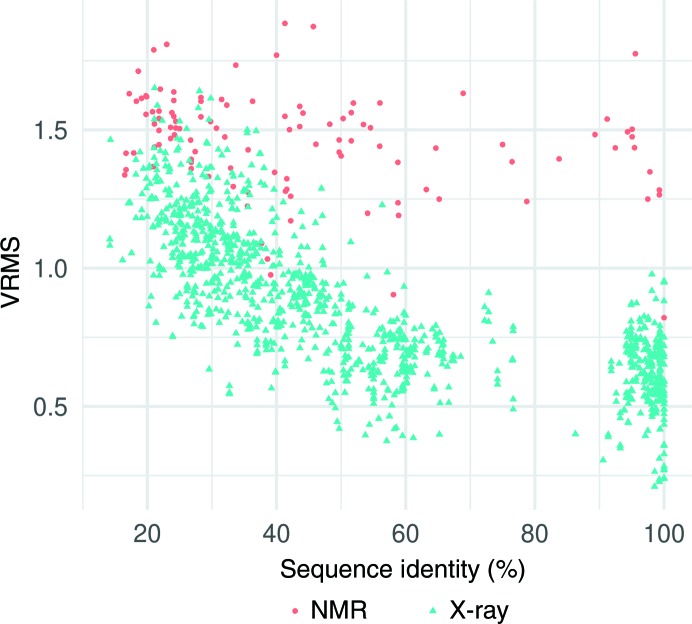
Comparative analysis of errors between X-ray and NMR models of size 150 ± 25 residues. Although the Gonnet score was used to estimate VRMS, sequence identity (*x* axis) is provided for ease of comparison.

Our error estimates show why molecular replacement with NMR models is a challenge, as NMR models have much higher estimated errors than comparable X-ray models. To compare model quality over the whole range of sequence identity, for structures of the typical size addressed by NMR, we supplemented our data set with all available models between 60 and 100% sequence identity for targets in our database of between 125 and 175 residues in size, adding 444 X-ray models and 20 NMR models. For this size range, we found that using an NMR model with 90–100% sequence identity is equivalent to using an X-ray model with about 20–30% sequence identity (Fig. 5 ▸). The data in this figure can be approximated reasonably well by assuming that the NMR models differ in having an additional independent error component with a standard deviation of about 1.25 Å. This error component dominates across the sequence-identity distribution.

## 4. Discussion   

The Oeffner estimation of VRMS for X-ray models was systematically overestimating the errors when the sequence identity was less than 30%. This artefact appears as a shoulder in the distribution of the ratio between refined and estimated VRMS (Figs. 3 and 5*b* in Oeffner *et al.*, 2013 ▸). Inspection of the cases populating this shoulder shows that this is owing to limitations in using sequence identity to measure sequence similarity between distant homologues.

After the target and model sequences have been optimally aligned, sequence identity represents a binary (true/false) score for each position in the alignment, which becomes a rather coarse measure for distant homologues with low sequence identity. Sequence identity also fails to distinguish between conservative and nonconservative substitutions. Hence, we considered 20 matrix scores, listed in Table 1 ▸ and discussed in the review by Vogt *et al.* (1995 ▸), which were expected to give a sensitive assessment of sequence similarity between homologues with less than 50% sequence identity. When we consider the full range of sequence identity (10–100%), BLOSUM30, BLOSUM35, BLOSUM40, BLOSUM45 (Henikoff & Henikoff, 1992 ▸), Benner22, Benner 74 (Bennet *et al.*, 1994 ▸) and Gonnet scores (Gonnet *et al.*, 1992 ▸) are all strongly correlated to the VRMS, with similar correlations of −0.70 to −0.71. Sequence identity gives a slightly weaker correlation of −0.67 (Table 2 ▸). However, looking at progressively lower levels of sequence identity, where MR is more challenging, some scoring matrices start to perform better. The Benner22, Benner74 and Gonnet scores all yield a correlation of −0.38 for models with sequence identity below 30%; for models with sequence identity below 20%, the Gonnet score gives a correlation of −0.15, which is slightly better than those of −0.14 for Benner74 and −0.11 for Benner22. Our observations agree with an earlier finding that the Gonnet score is one of the top three matrices to assess sequence similarity among distant homologues (Vogt *et al.*, 1995 ▸). By replacing sequence identity with the Gonnet score, we have addressed the systematic overestimation of errors in the distant homology regime.

While we were expecting to find a correlation to the resolution of the model, we were surprised to find target resolution instead to be correlated to the VRMS. Several other target properties such as asymmetric unit volume, Wilson *B* factor and Matthews coefficient are also correlated to the VRMS, but they are all correlated to each other and to the target resolution. Once the resolution of the target had been accounted for in the VRMS estimation, there were no residual correlations to these other target properties. This finding indicates that a higher r.m.s.d. should be expected if the crystal has diffracted to lower resolution. It could be explained by noting that crystals diffracting to lower resolution are intrinsically less well ordered and possess a larger number of conformational states, which are explained poorly by a single model. Similar conclusions have been drawn in the context of the gap between *R*_cryst_ and *R*_merge_ (Holton *et al.*, 2014 ▸).

Of the properties considered for evaluating model quality, resolution of the model, *R*_free_, clashscore and *MolProbity* score were all correlated with VRMS, with *MolProbity* score giving the highest correlation. These measures were all correlated to each other, and once the influence of *MolProbity* score had been accounted for there were no residual correlations with other properties of the model. Considering that *MolProbity* score (Chen *et al.*, 2010 ▸) combines contributions from clashscore, Ramachandran outliers and rotamer outliers, it is surprising that *MolProbity* score is a significantly better predictor than clashscore, even though the correlations with Ramachandran and rotamer outliers are small. This presumably indicates that *MolProbity* score nonetheless integrates the influence of all three measures to assess the quality of model building and refinement better than any of the measures on its own.

The properties correlated to VRMS in the case of X-ray models were also found to be correlated to VRMS for NMR models. However, the relative importance of these factors differs. For the X-ray case, the most important factors were sequence similarity measured by Gonnet score, followed by the number of residues in the model, the resolution of the target and the *MolProbity* score of the model. However, the number of residues in the model is the dominant factor for the NMR case with a correlation of 0.5, followed by Gonnet score, the resolution of the target and NMR ensemble consistency (measured as median r.m.s.d. between the models). Using the X-ray equation to estimate VRMS for NMR models will systematically underestimate the errors (Fig. 3 ▸), leading to suboptimal molecular-replacement calculations, so a separate nonlinear least-squares fit was performed for NMR models.

With the new functional forms, we have achieved better accuracy and a better (more symmetrical and unimodal) distribution of errors for the estimates. The new estimates perform better for both X-ray and especially for NMR models.

Representing and querying highly interconnected data as a graph simplifies data analytics. The graph database has enabled us to overcome redundancies in the data and has provided an easy way of extending the existing X-ray data along with the NMR data. It provided a platform to compare results from several trials of molecular-replacement runs quickly and consistently. Further extension of the data in the future, for example to include cryo-electron microscopy-related data, would also be possible.

By including properties of the target in the error estimates, we are pushing the boundaries of molecular replacement by personalizing the model for a given data set. The data-driven model generation will pave the way for handling complex molecular-replacement search strategies for structures with multiple domains or subunits.

The new VRMS estimates will be available as part of the *phaser.voyager* pipeline to run the new version of *Phaser*, *phasertng* (McCoy *et al.*, 2020 ▸), which is currently under development.

## Figures and Tables

**Table 1:** List of properties considered in the study The sequence-similarity measures have been discussed in a previous review (Vogt *et al.*, 1995 ▸) and citations therein. Ensemble consistency is measured as median r.m.s.d. between the models in an NMR ensemble.

Target properties	Model properties	Sequence-similarity measures
**Crystal parameters**: asymmetric unit volume, unit-cell dimensions, space group, Matthews coefficient, crystal system, polar space group	**Validation parameters**: Ramachandran properties, clashscore, rotamer outliers, *MolProbity* score, r.m.s.d. on angles, r.m.s.d. on bonds, C^β^ deviations, *R* factors†	Sequence identity, PAM250, PAM300, BLOSUM30, BLOSUM35, BLOSUM40, BLOSUM45, BLOSUM65, Benner6, Benner22, Benner74, Feng, Genetic, Gonnet, Johnson, Levin, McLach, Miyata, Rao, Risler, structure-based
**Data parameters**: resolution, Wilson *B* factor, merging statistics	**Data properties**: resolution†, completeness of resonance assignments‡, ensemble consistency‡, number of conformers deposited‡, number of conformers calculated‡, field strength‡	
**Protein properties**: number of residues, SCOP class	**Protein properties**: number of residues, molecular weight, nonsphericity, helix and sheet content	
	**Deposition date**	

† Properties specific to X-ray models.

‡ Properties specific to NMR models.

**Table 2:** Correlation of properties to the X-ray VRMS term Residual correlation is the correlation between the property and the difference between the estimated VRMS and the refined VRMS estimated either with the Oeffner equation (2) or the new equation (3).

Property	Correlation to VRMS	Residual correlation to VRMS	
		Oeffner estimate	New estimate
No. of residues of model	0.43	0.10	0.00
Sequence identity	−0.67 (−0.33†)	0.00	0.00
Gonnet score	−0.71 (−0.41†)	−0.16	−0.03
Target resolution	0.26	0.24	0.00
*MolProbity* score of model	0.16	0.18	−0.02
Percent α-helix	0.20	0.19	0.10
Percent β-sheet	−0.14	−0.16	−0.13

† Correlation for a subset of cases with <30% sequence identity

**Table 3:** Correlation of properties with VRMS for the case of NMR models Residual correlation is the correlation between the property and the difference between the estimated and refined VRMS terms.

Property	Correlation to VRMS	Residual correlation to VRMS	
		Oeffner X-ray estimate	New estimate
No. of residues of model	0.56	0.28	0.06
Gonnet score	−0.38	0.40	0.00
Target resolution	0.28	−0.05	−0.01
Median r.m.s.d.	0.22	0.14	0.02
*MolProbity* score of model	0.11	0.05	0.00
Percent α-helix	0.23	0.22	0.00
Percent β-sheet	0.07	0.24	−0.01
